# Supplementary figures and images for: Integration of sleep homeostasis and navigation in Drosophila
Source: PLoS Comput Biol. 2021 Jul 12;17(7):e1009088. doi: 10.1371/journal.pcbi.1009088 (PMC8297946; doi:10.1371/journal.pcbi.1009088)

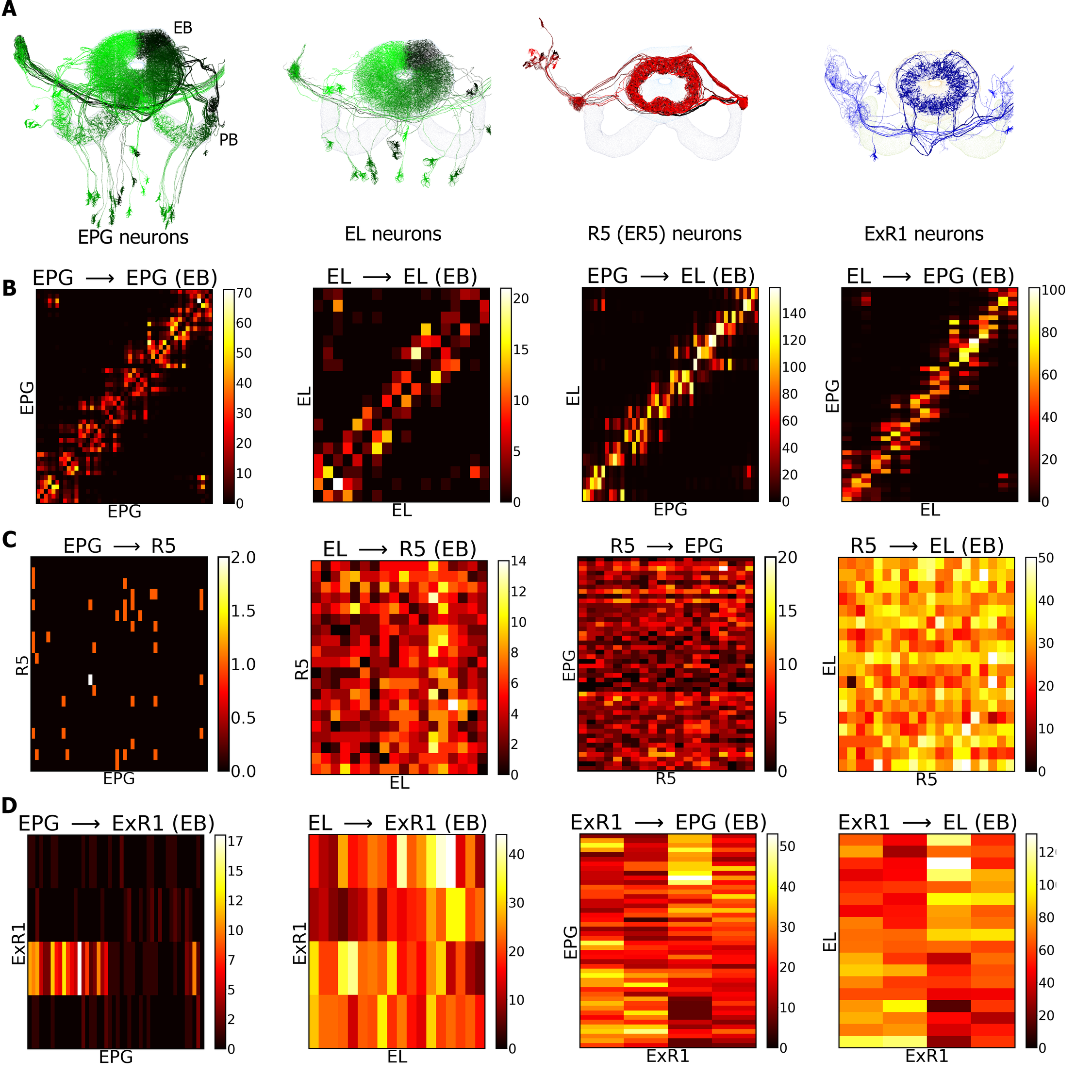

Supplement: S1 Fig — A: On the left, neural projections of EPG and EL, referred as wedge neurons (green). On the right, neural projections of R5 (red) and ExR1 (blue). B: Recurrent connectivity between wedge neurons. The matrix in each figure represents the number of synaptic sites between presynaptic neurons (horizontal axis) and postsynaptic neurons (vertical axis) [14]. C: Connectivity between wedge neurons and R5 neurons in both directions. D: Connectivity between wedge neurons and ExR1 neurons in both directions. Data and neurons are reproduced from [14]. (TIF) [file pcbi.1009088.s001.tif]

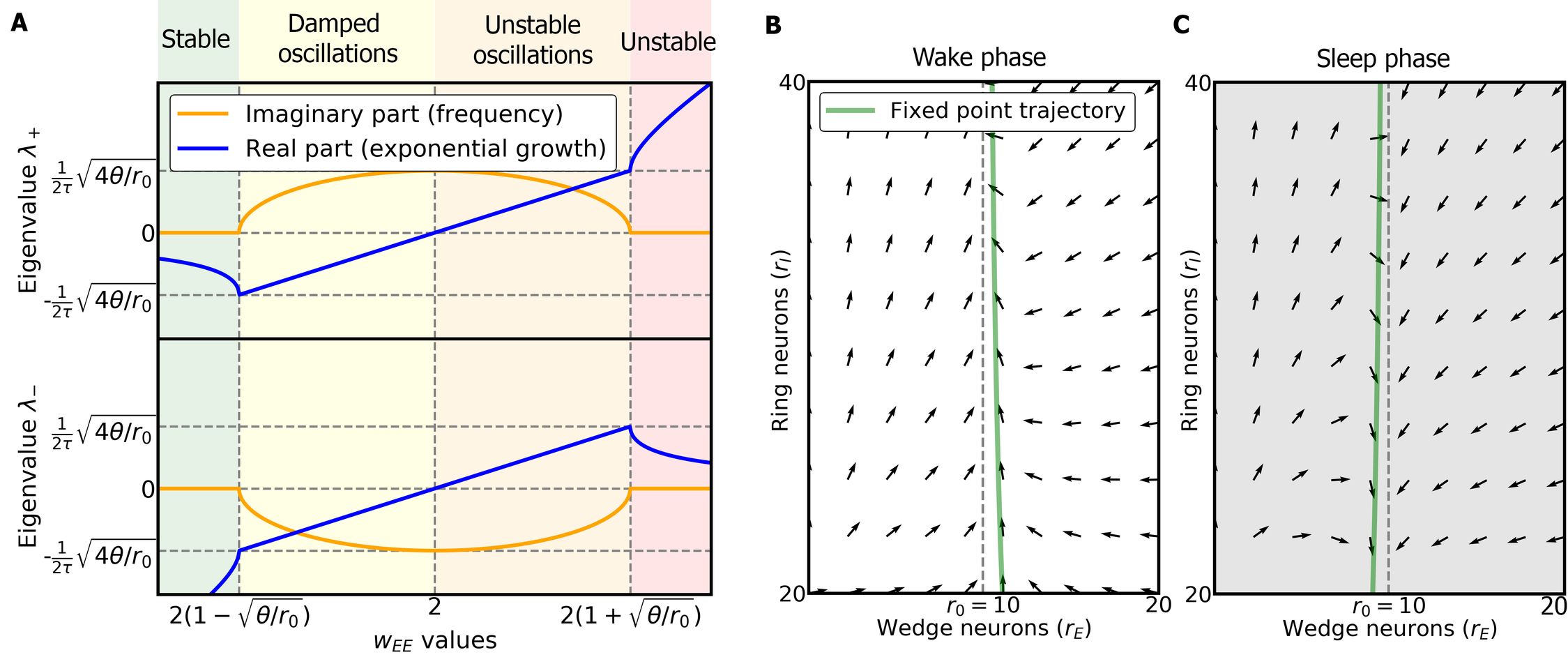

Supplement: S2 Fig — A: Eigenvalues in the two-population model in the fast-timescales limit. The real (in blue) and imaginary (in orange) parts of the eigenvalues are plotted as a function of wEE. B: Vector field of wedge and ring neurons dependent on synaptic plasticity during the wake phase in the slow-timescale limit. The green line represents the set point trajectory of wedge neurons. C: Vector field of wedge and ring neurons due to plasticity in the sleep phase in the slow-timescale limit. The green line is the trajectory of the set point in wedge neurons. (TIF) [file pcbi.1009088.s002.tif]

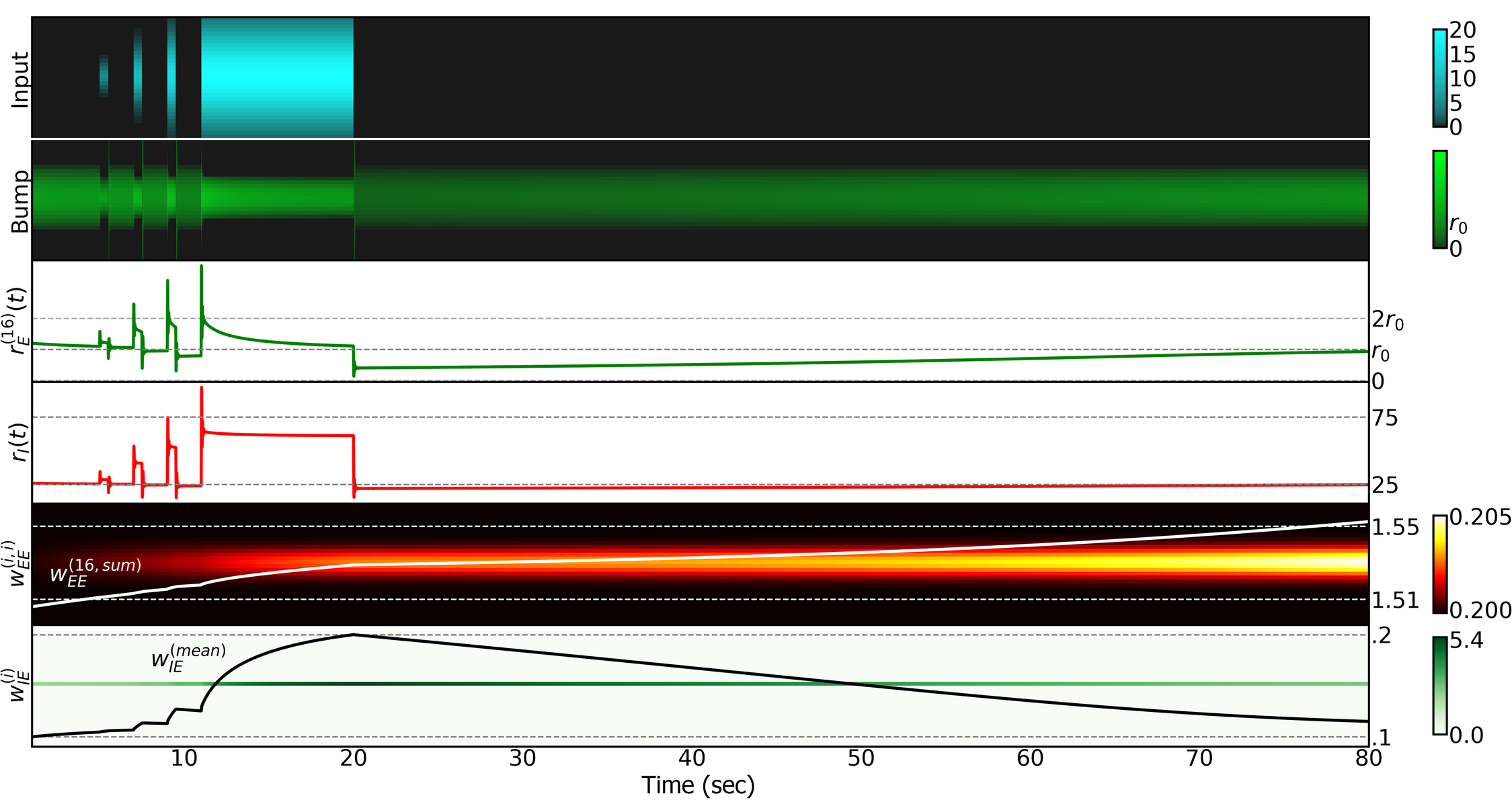

Supplement: S3 Fig — When input (first row) is provided to the ring attractor the plasticity rule for wIE(i) brings the activity of the wedge neuron rE(16) (where the bump peak is located, second row) back to r0 (third row). The bump is not constrained to have constant activity (third row), but always relaxes towards r0 over time in the absence of changing input. For ease of visualization, we used a slower time constant for the plasticity of wEE(ij) (τEE = 1000000), thus avoiding oscillations in the bump throughout the simulation. (TIF) [file pcbi.1009088.s003.tif]

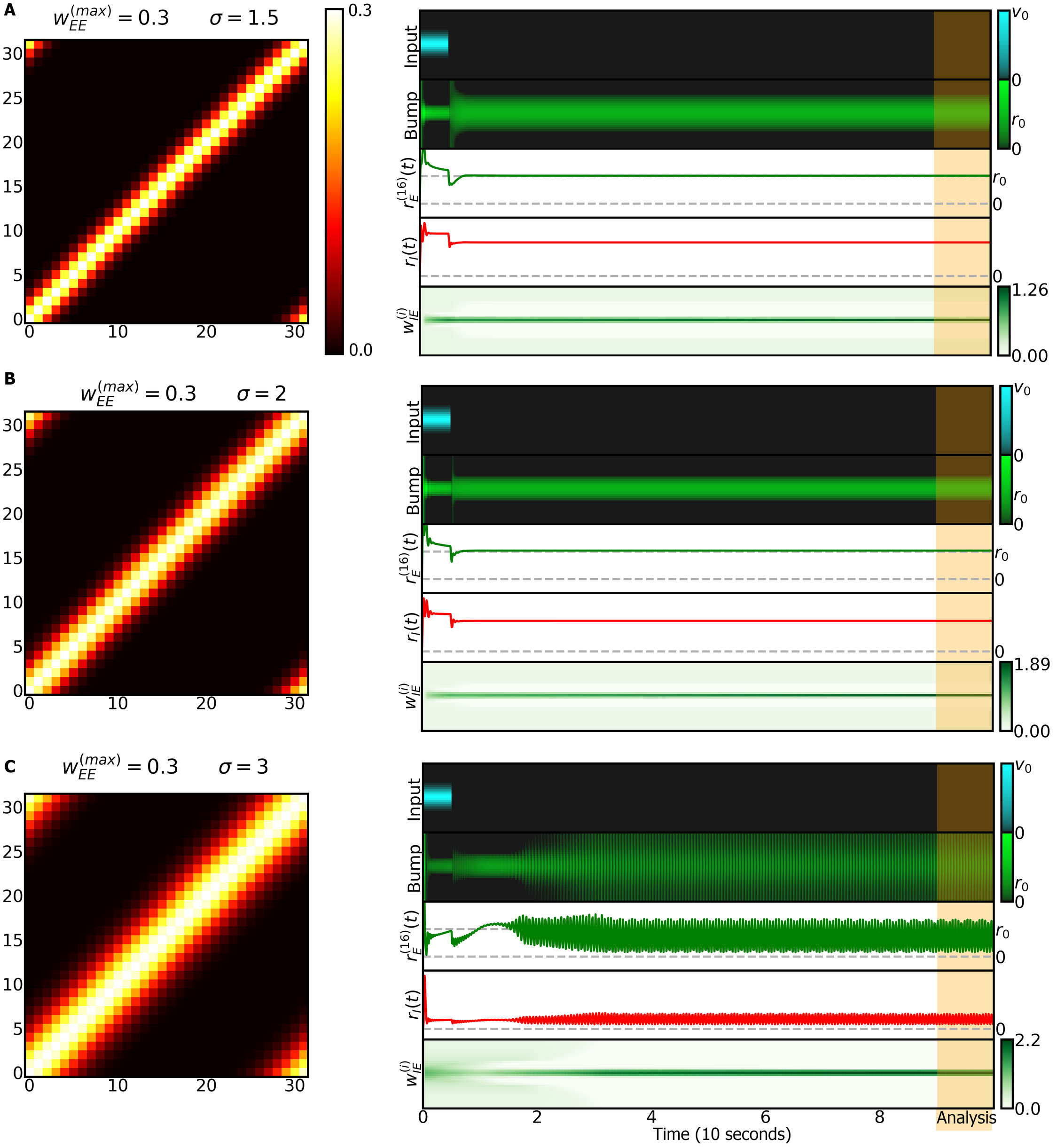

Supplement: S4 Fig — A: Left: initial wEE(ij) values; right: 10 seconds of simulated dynamics. First row: input positioning the bump around wedge neuron 16. Second row: bump profile over time. Third row: activity of wedge neuron 16 evolving towards r0 due to plasticity in wIE(i). Fourth row: activity of ring neurons. Fifth row: the synaptic weights wIE(i). The orange band at the end of the simulations represents the period in which the stability of the bump is analyzed. B: Same as A but with larger σ. C: Same as A, B but again increasing σ. In this simulation, the bump shows stable oscillations. (TIF) [file pcbi.1009088.s004.tif]

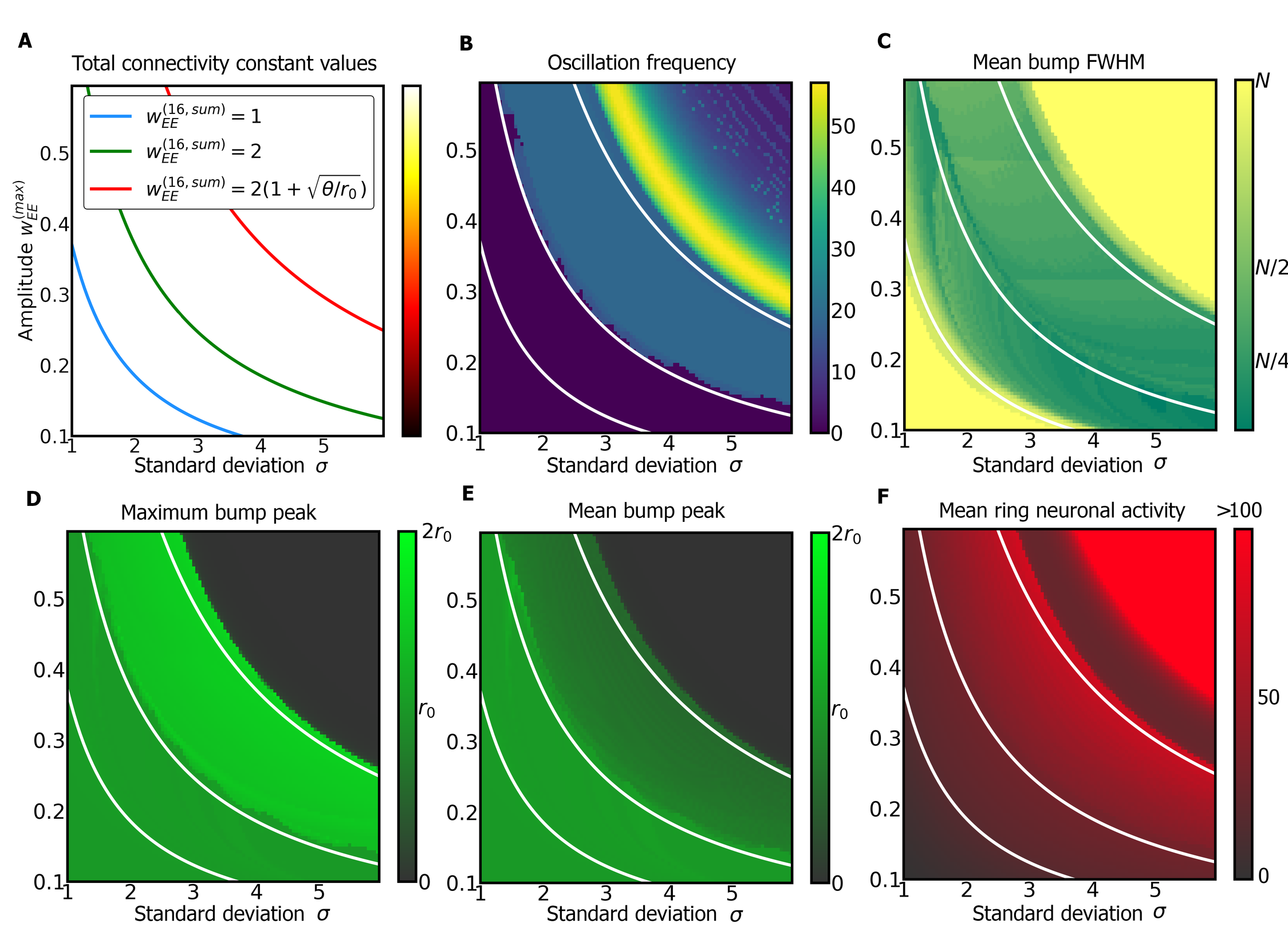

Supplement: S5 Fig — All subfigures are plots of different measures as a function of σ and wEE(max), which parameterize the initial synaptic weights wEE(ij) (Methods (4.7)). All variables in B, C, D, E, and F are calculated in a time window of 1 sec at the end of each simulation (orange area in Fig 3). A: Isolines of wEE(16,sum). These isolines are overlaid in white in the other subfigures and represent approximate boundaries of stability. B: Frequency of the oscillations in the bump across the analysis time window. Above the isoline wEE(16,sum)=2, the bump oscillates. C: Mean FWHM over the analysis time window. Below the isoline wEE(16,sum)=1 and above the isoline wEE(16,sum)=2(1+θ/r0), there is no bump because the FWHM is equal to the number of neurons, N. D: Maximum peak of the bump in the analysis time window (maximum activity of wedge neuron 16, max(rE(16)(t))). Below the isoline wEE(16,sum)=2, the maximum is r0 as forced by the plasticity rule in wIE(i). Between the isolines wEE(16,sum)=2 and wEE(16,sum)=2(1+θ/r0), the bump oscillates and the amplitude is given by the maximum, in this case 2r0. Above wEE(16,sum)=2(1+θ/r0), there is close-to-zero activity in wedge neurons, since the maximum is near zero. E: Mean of the bump peak over the analysis time window (mean activity of the wedge neuron 16, <rE(16)(t)>). In case of no oscillations, this value should be equivalent to the maximum bump peak in panel D. During oscillations, the value closely represents the center of oscillations. Note that between isolines wEE(16,sum)=2 and wEE(16,sum)=2(1+θ/r0) this value is lower than r0. F: Mean activity over the analysis time window of ring neurons, <rI(t)>. Below the isoline wEE(16,sum)=2(1+θ/r0), the activity of ring neurons increases. Above the isoline, the activity of ring neurons first decreases and then rapidly increases. We clipped the values of the mean activity above 100 to facilitate visualization, but the increment of activity in this area reached values over 1000. (TIF) [file pcbi.1009088.s005.tif]

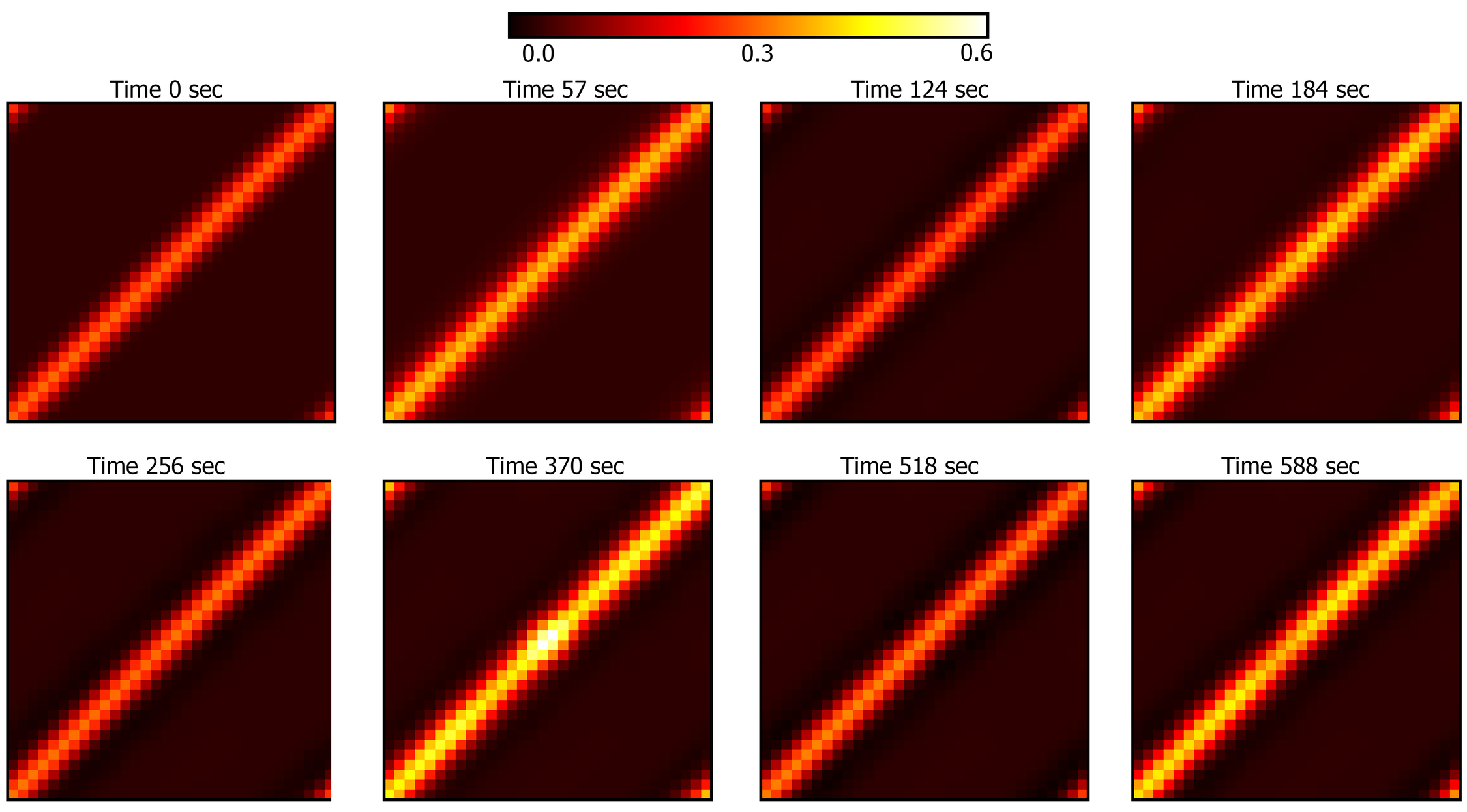

Supplement: S6 Fig — From left to right and from top to bottom: each plot shows the values each time there is a switch between wake and sleep phase and vice versa. Top, left: initial values of the weights set by the initial values of wEE(max) and σ in Table B in S1 Text. Top, center left: weights at the end of the first wake phase. Top, center right: after the subsequent sleep phase. Top, right: after the second wake phase. Bottom, left: weights after the second sleep phase. Bottom, center left: after sleep deprivation. Bottom, center right: after sleep rebound. Bottom, right: weights after the third wake phase. Note how the weights after each wake phase are increased (specially after sleep deprivation in the sixth plot) and how the sleep phase resets the weights to close to the initial conditions. (TIF) [file pcbi.1009088.s006.tif]

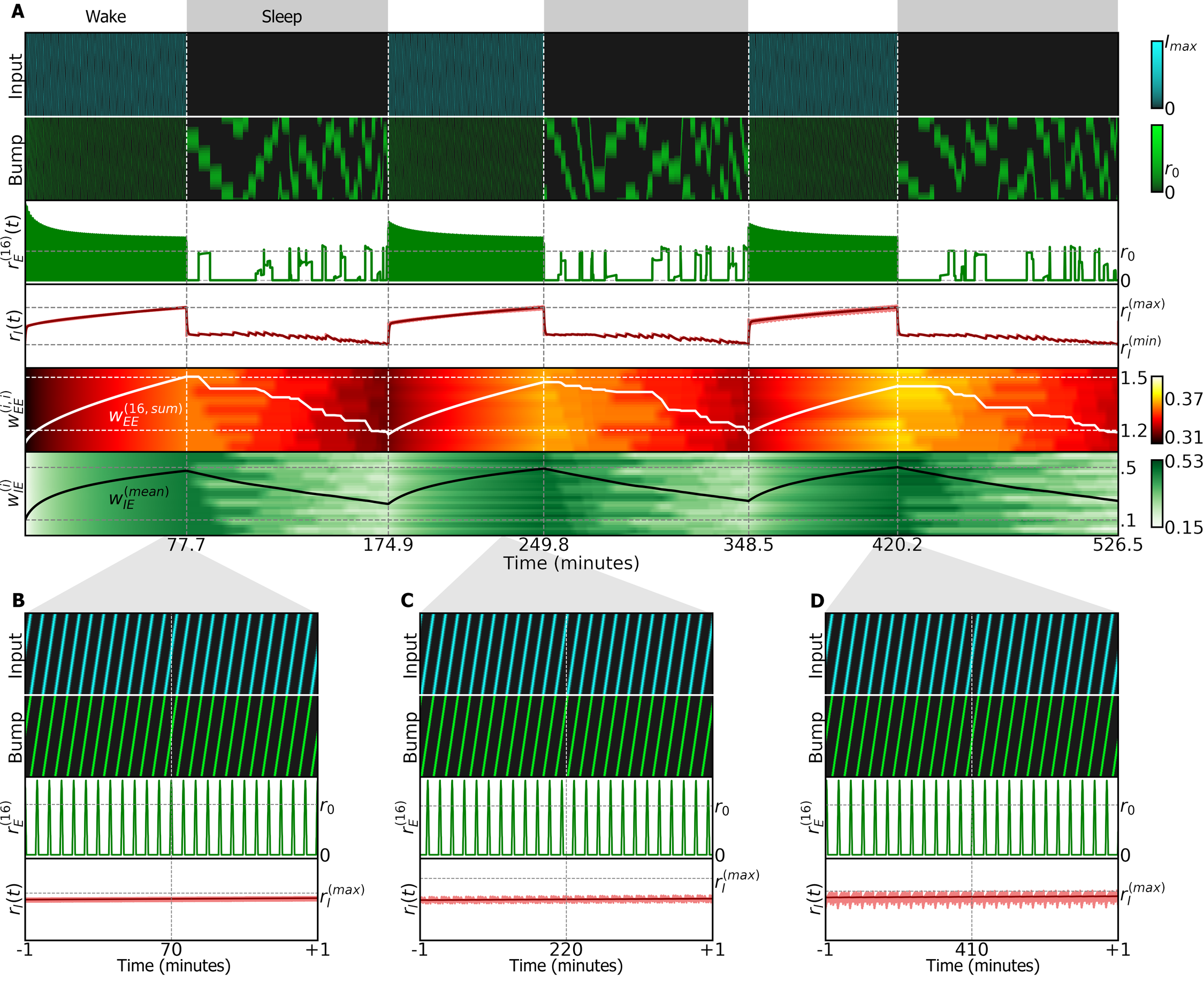

Supplement: S7 Fig — The time constants τEE and τIE in this simulation are 210 times larger than in Table B in S1 Text. We decreased the time resolution of the simulation (time step is 0.001 seconds) and decreased the value rI(max) to 30. A: Full simulation with three wake phases and three sleep phases. We used the same input rotation as in Fig 5 (rotating input at 0.5Hz). B: Blow-up around 70 minutes in the first wake phase of the simulation. C: Blow-up around 220 seconds in the second wake phase. D: Blow-up around 410 seconds in the third wake phase. Note that the duration of sleep and wake phases are now on the order of hours due to the increased time constants in the plasticity rules. (TIF) [file pcbi.1009088.s007.tif]

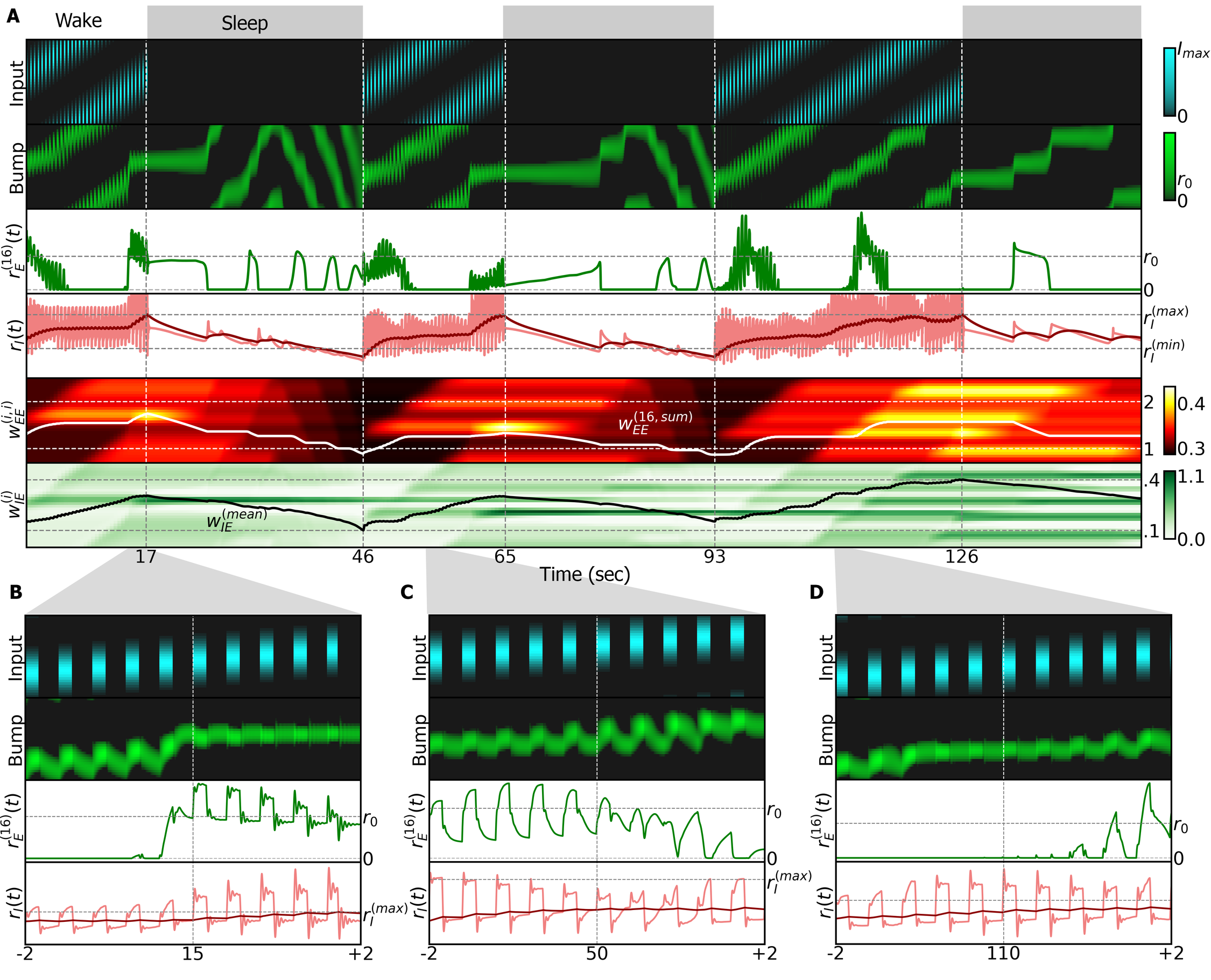

Supplement: S8 Fig — A: Simulation with three wake phases and three sleep phases. A flashing input that turns on and off is provided during the wake phase. During the off period, the ring attractor sustains a bump of activity that drifts due to changes in the synaptic weights wEE(ij). B: Blow-up around 15 seconds in the first wake phase of the simulation. C: Blow-up around 50 seconds in the second wake phase. D: Blow-up around 110 seconds in the third wake phase. (TIF) [file pcbi.1009088.s008.tif]

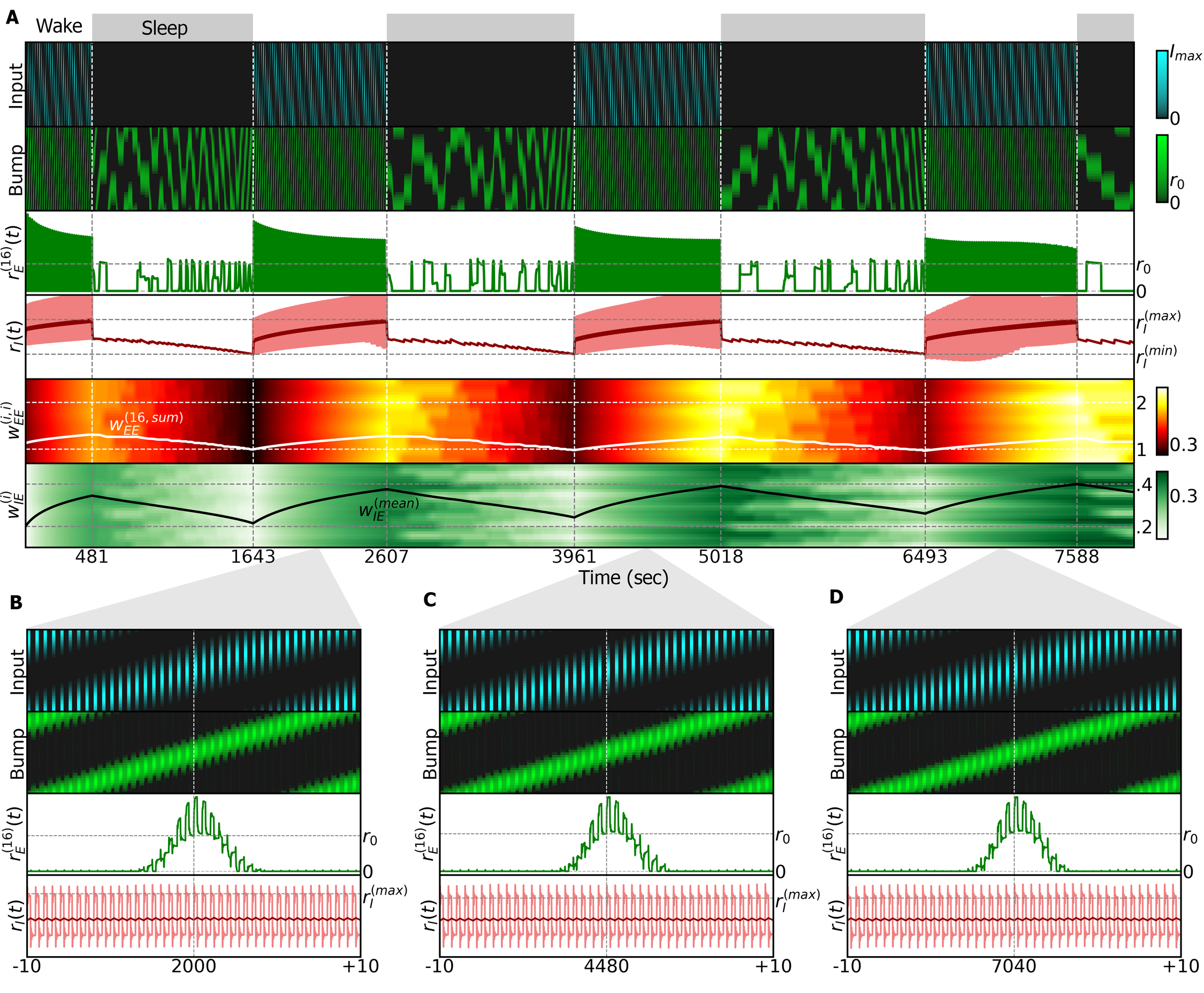

Supplement: S9 Fig — The time constants in this simulation are 100 times larger than in the previous ring attractor simulations. A: Full simulation with three wake phases and three sleep phases. We used the same input rotation frequency as in S6 Fig. As in S6 Fig, we provide a flashing input that turns on and off during the wake phase. B: Blow-up around 2000 seconds in the first wake phase of the simulation. C: Blow-up around 4480 seconds in the second wake phase. D: Blow-up around 7040 seconds in the third wake phase. Note how the duration of sleep and wake phases are now on the order of hours due to the increased time constants in the plasticity rules. Note also how the bump of activity is sustained in place without drifting after the input switches off. (TIF) [file pcbi.1009088.s009.tif]

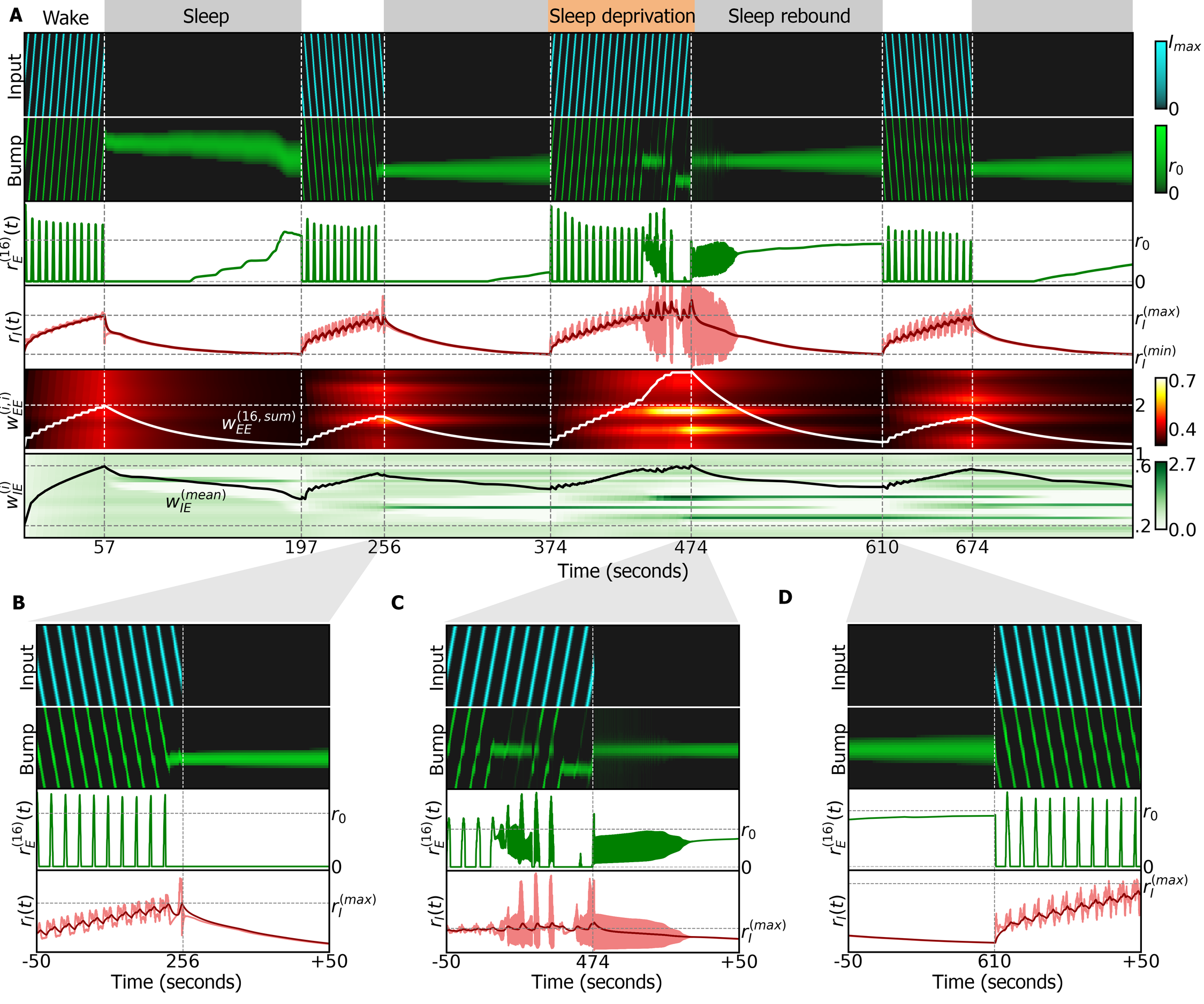

Supplement: S10 Fig — A: Entire simulation over a period of 800 seconds. White and grey regions indicate the sleep and wake phases, and correspond to dFB neurons switching off and on, respectively. Top row: input (inhibited during the sleep phase), alternating between clockwise and counter-clockwise rotations at 0.5Hz. Second row: ring attractor bump activity. Third row: activity of wedge neuron 16. Fourth row, light red: activity of R5 neurons. Dark red: filtered activity. Switching between sleep and wake is carried out by dFB neurons that switch on and off depending on filtered activity crossing thresholds rI(min) and rI(max). In the third wake epoch, sleep deprivation is produced by extending the inhibition of dFB neurons (d(t) = 1 during the orange top layout). Fifth row: diagonal elements of the connectivity matrix wEE(ij). The white line is the sum of all excitatory connections to wedge neuron 16. It passes threshold 2 at around 240 seconds leading to oscillations. The full connectivity matrix wEE(ij) at the switch times is shown in S6 Fig. Sixth row: connectivity wIE(i); black line is the mean value. B: Blow-up around 256 seconds: switch from wake to sleep phase. C: Blow-up around 474 seconds: extended wake phase leads to oscillatory behavior. Circuit switches to sleep. C Blow-up around 610 seconds: switch from sleep to wake phase. (TIF) [file pcbi.1009088.s010.tif]
