# Supplementary material for: Integration of sleep homeostasis and navigation in Drosophila
Source: PLoS Comput Biol. 2021 Jul 12;17(7):e1009088. doi: 10.1371/journal.pcbi.1009088 (PMC8297946; doi:10.1371/journal.pcbi.1009088)
Supplement: S1 Text — Table A shows parameter values used in the two-population model, Table B shows parameter values used in the ring attractor network and a model for ring attractor network resetting of weights during sleep without autonomous activity is presented. (PDF) [file pcbi.1009088.s011.pdf]

S1 Text

τ	τ_f	c	τ_{EE}	τ_{IE}	w_{EI}	θ	r_0	$r_I^{(min)}$	$r_I^{(max)}$
0.01	2	0.005	50	50	0.5	10	10	20	35

Table A: **Parameter values used in the two-population model.** Time constants τ, τ_f, τ_{EE} and τ_{IE} are measured in seconds. We consider the rest dimensionless quantities.

τ	τ_f	c	τ_{EE}	τ_{IE}	w_{EI}	θ	r_0	$r_I^{(min)}$	$r_I^{(max)}$	$w_{EE}^{(max)}$	σ	I_{max}	I_σ
0.01	2	0.005	50	50	0.5	10	10	22	30	0.3	1.5	5	2

Table B: **Parameter values used in the ring attractor network.** As in the previous model, time constants τ, τ_f, τ_{EE} and τ_{IE} are measured in seconds. We consider the rest dimensionless quantities.

Ring attractor network: resetting the weights during sleep without autonomous activity

Assuming anti-Hebbian plasticity in the ring attractor network during sleep leads to autonomous dynamics (section 2.7). A different way to reset the network connectivity $w_{EE}^{(ij)}(t)$ is to relax it to its initial values over the sleep phase:

$$\tau_{EE} w_{EE}^{(ij)}(t) = -w_{EE}^{(ij)}(t) + w_{EE}^{(ij)}(0) \qquad for \qquad i, j = 1, ..., N. \tag{1}$$

S10A Fig shows a simulation of the ring attractor model using this type of plasticity during sleep. blackDuring the wake phase the dynamics of the model does not change (top, white regions). However, during sleep the plasticity rule keeps the bump in wedge neurons in place (top, grey regions), maintaining the last head direction of the fly before the sleep phase while the activity of R5 neurons slowly decreases. As with the two-population model (Methods 4.5), the timescale of sleep and wake phases depends on the time constants of the plasticity rules.
